# Transition metal atom doping of the basal plane of MoS_2_ monolayer nanosheets for electrochemical hydrogen evolution†
†Electronic supplementary information (ESI) available. See DOI: 10.1039/c8sc01114a


**DOI:** 10.1039/c8sc01114a

**Published:** 2018-04-30

**Authors:** Thomas H. M. Lau, XiaoWei Lu, Jiří Kulhavý, Simson Wu, Lilin Lu, Tai-Sing Wu, Ryuichi Kato, John S. Foord, Yun-Liang Soo, Kazu Suenaga, Shik Chi Edman Tsang

**Affiliations:** a Department of Chemistry , University of Oxford , Oxford , OX1 3QR , UK . Email: Edman.tsang@chem.ox.ac.uk; b College of Chemistry and Chemical Engineering , Wuhan University of Science and Technology , China; c Department of Physics , National Tsing Hua University , Hsinchu , Taiwan; d National Institute of Advanced Industrial Science and Technology (AIST) , Central 5, 1-1-1 Higashi , Tsukuba , Ibaraki 305-8565 , Japan

## Abstract

Surface sites of extensively exposed basal planes of MoS_2_ monolayer nanosheets, prepared *via* BuLi exfoliation of MoS_2_, have been doped with transition metal atoms for the first time to produce 2D monolayer catalysts used for the electrochemical hydrogen evolution reaction (HER). Their HER activity is significantly higher than the corresponding thin and bulk MoS_2_ layers. HAADF-STEM images show direct proof that single transition metal atoms reside at the surface basal sites, which subtly modify the electro-catalytic activity of the monolayer MoS_2_, dependent on their electronic and stereospecific properties. It is found that these dopants play an important role in tuning the hydrogen adsorption enthalpies of the exposed surface S atoms and Mo atoms in HER. We report electrochemical testing, characterization and computational modelling and demonstrate that Co can significantly enhance the HER activity by the dominant Co–S interaction, whereas Ni substantially lowers the HER rate due to the Ni–Mo interaction at the same basal site. The two transition metal dopants show opposite doping behavior despite the fact that they are neighbors in the periodic table.

## Abstract 2

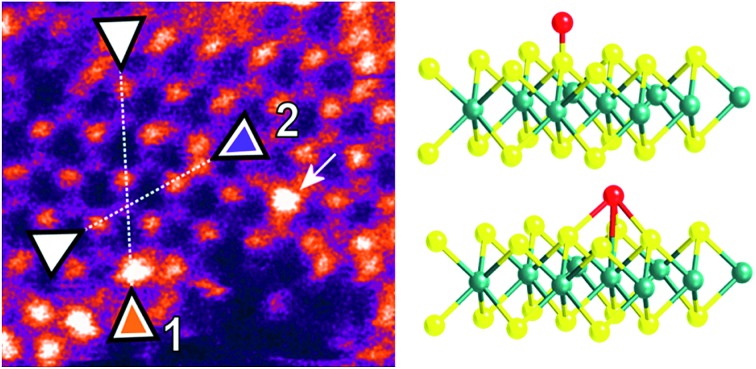
A Co atom enhances the HER activity of monolayer MoS_2_ whereas a Ni atom exhibits the opposite effect on the same basal site.

## Introduction

There is an increasing demand for hydrogen to play a larger role in enabling the use of renewable energy sources (*i.e.* solar, wind, hydropower *etc.*) to reduce the carbon emissions from various industries in the near future. Particularly, the progressive decrease in the cost of electrolysers and the possible implementation of carbon taxation may justify large scale H_2_ production from the electrolysis of water in centralised installations. However, noble metals are commonly used as catalysts for both the anode and cathode in the electrolytic production of hydrogen from water. Their high cost is currently prohibitive in scaling up green hydrogen production. Therefore, developing inexpensive and Earth-abundant catalytic materials for the electrolytic hydrogen production reaction (HER) is of great interest. The discovery of graphene demonstrated that the material properties of an atomically thin material can be fairly distinct from the bulk counterpart.^1^ This has stimulated the extensive exploration of different two-dimensional (2D) materials. This research therefore stems from the current interest in layered materials, which represent a diverse and largely untapped source of 2D systems. As one of the emerging layered materials, 2D molybdenum disulphide (MoS_2_) has drawn growing research attention in recent years due to its novel electronic, optical, mechanical, and electrochemical properties that are important for sensing,^2^,^3^ catalysis,^4^,^5^ and energy storage applications.^6^,^7^

A single tri-atomic MoS_2_ layer has a chemical structure of a hexagonal plane of molybdenum atoms sandwiched between two other hexagonal planes of sulphur atoms *via* strong in-plane covalent bonding. Adjacent layers are then held by weak out-of-plane van der Waals interactions.^8^ It is well accepted that the Mo edges (101 It is well accepted that the Mo edges (101̄0) and the S edges (1̄010) of the MoS0) and the S edges (1 It is well accepted that the Mo edges (101̄0) and the S edges (1̄010) of the MoS010) of the MoS_2_ particles provide the two main active sites for most electrochemical reactions, including HER.^9^ They are preferentially exposed for reaction, which is one of the reasons why 2D MoS_2_ shows overall better performance in electrochemical catalysis. In contrast, the basal plane (0001) has been verified to be electrochemically inert^10^ and thus is usually ignored as a contributor to the catalytic activity. In an attempt to enhance the activity of MoS_2_ for HER, the general strategies are to increase the density of these active edge sites by tuning and exposing more edge planes to the surface^11^–^14^ or using doper promoters^15^ to improve the conductivity and binding energy to hydrogen of the active sites. It wasn’t until recently that the presence of S vacancies as surface defects was shown to be able to activate the basal plane of MoS_2_, which rendered the material highly active for hydrogen evolution reaction (HER).^16^ To further enhance the exposure of the basal planes, exfoliation of bulk MoS_2_ may be performed to decrease the thickness of the planar layers by solvent exfoliation^8^,^17^,^18^ and Li exfoliation^19^,^20^ such that the interlayer van der Waals forces are partially or totally overcome, respectively. This opens up a new research area of studying and engineering the basal plane active sites of MoS_2_.^21^–^23^

There have been some testing and theoretical studies using bulk or thin MoS_2_ layers with and without metal promotion for HER, but not much work on employing a single MoS_2_ layer for modification at the atomic level for the HER reaction.^24^–^28^ Thus, the direct experimental proof of high quality samples with a single transition atom doped on monolayer MoS_2_ and correlation to HER activity is not yet established, despite some excellent images of single atoms on thin MoS_2_ layers.^28^ Additionally, the fundamental modification mechanisms of the single transition metal dopants on monolayer MoS_2_ for HER are not yet clear. Herein, we report our systematic experimental and modelling approaches to investigate high quality exfoliated single layer ^S^MoS_2_ samples, with and without transition metal doping, as 2D catalysts for electrochemical hydrogen production, which we believe can offer comprehensive insight into the structure–activity relationship of the 2D monolayer type of catalyst. Particularly, the bonding sites of Co and Ni on the basal planes of the MoS_2_ nanosheets (Co–^S^MoS_2_ and Ni–^S^MoS_2_) are directly imaged by HADDF-STEM and probed by XAS spectroscopy. It is evident that the use of different transition metal atoms as promotors can greatly influence the electronic properties and HER catalytic activity of the metal doped MoS_2_ nanosheets due to the geometric characteristics and the characteristic binding affinities of the transition metals to the surface sites. Typically, it is found that Co displays a high affinity to bind on exposed surface S sites but is unable to bind on Mo sites (no Co–Mo interaction), whereas Ni can give rise to an Ni–Mo interaction due to stereo-specificity in the metal–metal bond formation. Density functional theory calculations were also employed to assess the thermodynamic favourability of the doping processes. It is believed that through this investigation, more insight can be provided into elucidating the effect of single atomic dopants on 2D layered materials to enable new catalytic or electrocatalytic reactions.

## Results and discussion

### Catalytic performance and electronic properties

**Fig. 1:**
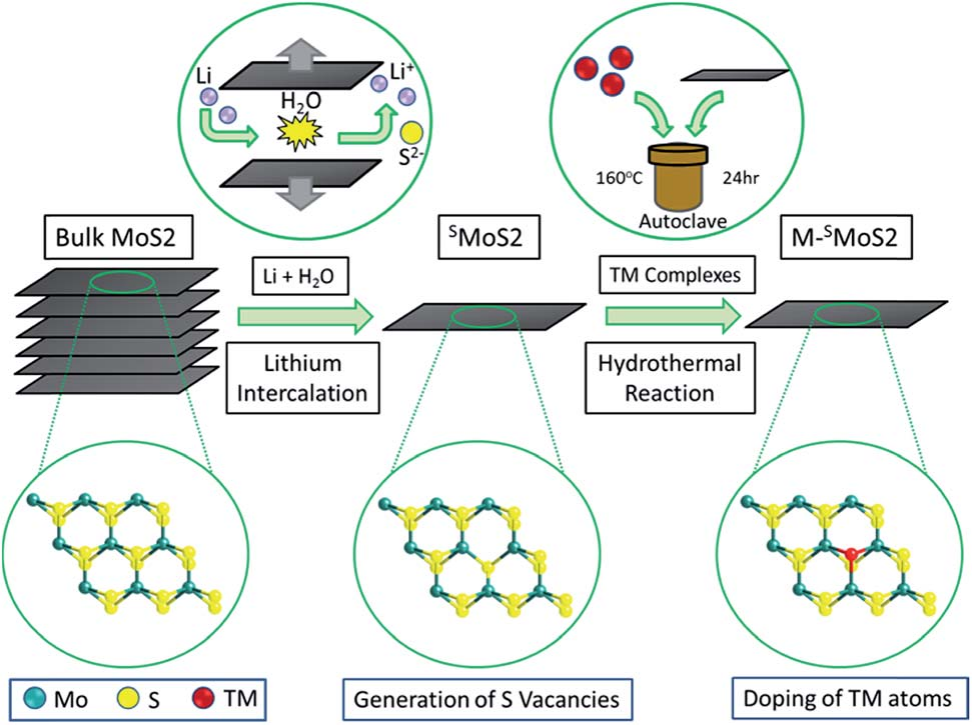
Synthesis of single-layered MoS_2_ (^S^MoS_2_) and single transition-metal (TM) atom doped ^F^MoS_2_/^S^MoS_2_SO_4_^2–^ groups.

The monolayer molybdenum disulfide nanosheets (^S^MoS_2_) were prepared *via* lithium intercalation from a bulk molybdenum disulfide precursor.^29^,^30^ (Fig. 1, synthetic details are provided in the ESI†). The sheet-like morphology of exfoliated MoS_2_ with regions of 1–3 molecular layers was characterized by high resolution transmission electron microscopes. Atomic force microscopy enabled the statistical analysis of 100 flakes produced by the lithium exfoliation method, which showed that 56% of the flakes were monolayer, 28% had two layers and 13% had three layers, with a lower concentration of the thicker flakes. (ESI, Fig. S1 and S2†). Different single transition metal atom doped MoS_2_ nanosheets (M–^S^MoS_2_, where M = Fe, Co, Ni and Ag) were then synthesised from ^S^MoS_2_ through a hydrothermal doping method. The details of the preparation and material characterization are summarized in S1 and S2 in the ESI .†

**Fig. 2:**
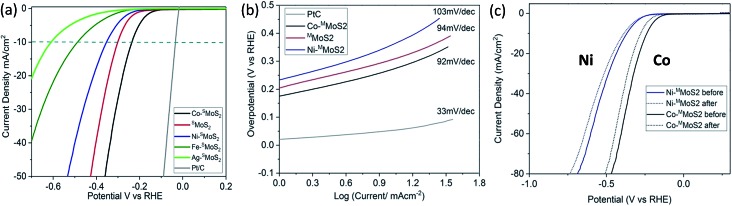
HER activity analysis by linear sweep voltammography, LSV. (a) LSV of the M–^S^MoS_2_ nanosheets and the reference 20% Pt/C in 0.5 M H_2_SO_4_ at a scan rate of 2 mVs^–1^. (b) Tafel plot of Co–^S^MoS_2_, ^S^MoS_2_, Ni–^S^MoS_2_ and 20%Pt/C. (c) LSV of Co–^S^MoS_2_ and Ni–^S^MoS_2_ before and after 1000 repeat scans at a scan rate of 50 mV s^–1^.

The electrochemical HER activity of the M–^S^MoS_2_ nanosheets was measured with pre-calibration (ESI, Fig. S3a†) and is shown in Fig. 2a. From the linear sweep voltammogram (LSV), the ^S^MoS_2_ nanosheets exhibit a distinctive HER catalytic activity when decorated with different single metal atoms. Among the tested samples, Co–^S^MoS_2_ appears to significantly promote the activity of the ^S^MoS_2_ nanosheets with the lowest onset potential of 220 mV at 10 mA cm^–2^. Taking this sample as a reference, Co atom doping on ^S^MoS_2_ nanosheets prepared by our Li intercalation route is significantly more active (lowest onset potential) than corresponding few layer ^F^MoS_2_ and bulk MoS_2_ samples (Fig. S3b†). This suggests that the activity is highly dependent on the quality of the MoS_2_ used. Previous studies in the literature using thin or few MoS_2_ layers with and without metal promotion for HER might have underestimated the metal promotion effect on single basal MoS_2_ with defects on the terrace surface. Interestingly, other transition metal doped nanosheets follow the order Ag < Fe < Ni, and show a higher onset potential than pristine ^S^MoS_2_ (300 mV at 10 mA cm^–2^). Typically, Ni–^S^MoS_2_ has an onset potential of 353 mV at 10 mA cm^–2^. The data clearly suggests that the choice of transition metal dopant greatly influences the HER catalytic performance of the basal ^S^MoS_2_ materials. It is known that the effect of the metal dopant is to modify the H adsorption enthalpy of the surface edge S sites and Mo sites in HER.^31^ It would be interesting to see whether the basal sites can be modified by the metal dopants and their effect on the HER. In addition, Ni and Co are two neighbouring transition metal elements in the periodic table. One would expect their electronic effects to be very similar to each other.^32^,^33^ Clearly, our HER measurements cannot be simply accounted for by a volcano plot,^34^ indicating that the HER activity may also be affected by other factors.

To elucidate this relationship, Ni–^S^MoS_2_ and Co–^S^MoS_2_ were purposefully selected for comparison. Tafel analysis was performed on both materials to understand the inherent electrochemical HER reaction mechanism, as shown in Fig. 2b. The Tafel slopes of Ni–^S^MoS_2_ and Co–^S^MoS_2_ have close values of 103 mV dec^–1^ and 92 mV dec^–1^, respectively. Since both values are similar to that of pristine ^S^MoS_2_ (94 mV dec^–1^), this suggests that all three MoS_2_ nanosheets follow the same HER reaction mechanism, *i.e.* the Volmer–Heyrovsky mechanism.^35^ An electrochemical durability test was also carried out to analyse the doping stability of the two nanosheet samples. Fig. 2c shows a comparison of the catalytic performances of Co–^S^MoS_2_ and Ni–^S^MoS_2_ before and after 1000 scans. Both nanosheets retain most of their HER activity, which indicates that the metal atoms were firmly attached onto the ^S^MoS_2_.

### Chemical and structural characterisation of M–^S^MoS_2_

In general, doping metals on a MoS_2_-based nanosheet increases the HER activity.^35^,^36^ For example, Ni nanoparticle doped MoS_2_ films were found to perform (onset potential: 310 mV at 10 A cm^–2^) better than the pristine film (onset potential: 350 mV at 10 A cm^–2^).^37^ However, the metal dopants are in the form of clusters or nanoparticles. They can give excellent electrocatalytic activity on their own while the 2D MoS_2_ this time serves as a supporting substrate.

As seen from the TEM images (ESI†), we did not see any Co or Ni containing nanoparticles on the MoS_2_ sheets. Inductively coupled plasma mass spectrometry (ICP-MS) was employed to analyse the concentration of the M–^S^MoS_2_ surface (ESI, Table S1†). A typical result shows a Co metal content (mg mg^–1^) of around 3.0 wt% and a corresponding calculated [Co/Mo] ratio of 11 can thus be deduced for M–^S^MoS_2_. Thus, the hydrothermal synthesis process employed using transition metal precursors in thiourea is proven to be successful in immobilizing metal dopants on the ^S^MoS_2_ surface.

**Fig. 3:**
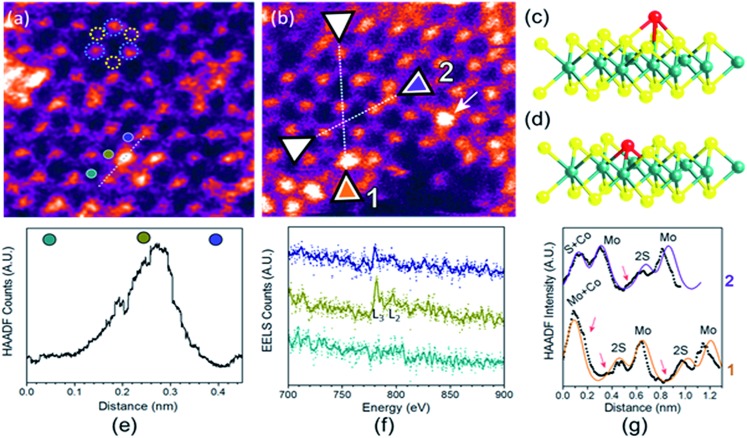
(a) and (b) HAADF-STEM images of Co–^S^MoS_2_.^29^ Simultaneous acquisition of (c) Co at the Mo-atop site model and (d) Co at the S vacancy site model (e) ADF and (f) EELS acquired along the line in (a). (g) ADF intensity line profiles taken along the numbered lines 1 and 2 shown in (b) ( Simultaneous acquisition of (c) Co at the Mo-atop site model and (d) Co at the S vacancy site model (e) ADF and (f) EELS acquired along the line in (a). (g) ADF intensity line profiles taken along the numbered lines 1 and 2 shown in (b) (〈110〉 direction). The red arrows in the plot indicate sample drift during image acquisition.110 Simultaneous acquisition of (c) Co at the Mo-atop site model and (d) Co at the S vacancy site model (e) ADF and (f) EELS acquired along the line in (a). (g) ADF intensity line profiles taken along the numbered lines 1 and 2 shown in (b) (〈110〉 direction). The red arrows in the plot indicate sample drift during image acquisition. direction). The red arrows in the plot indicate sample drift during image acquisition.

**Fig. 4:**
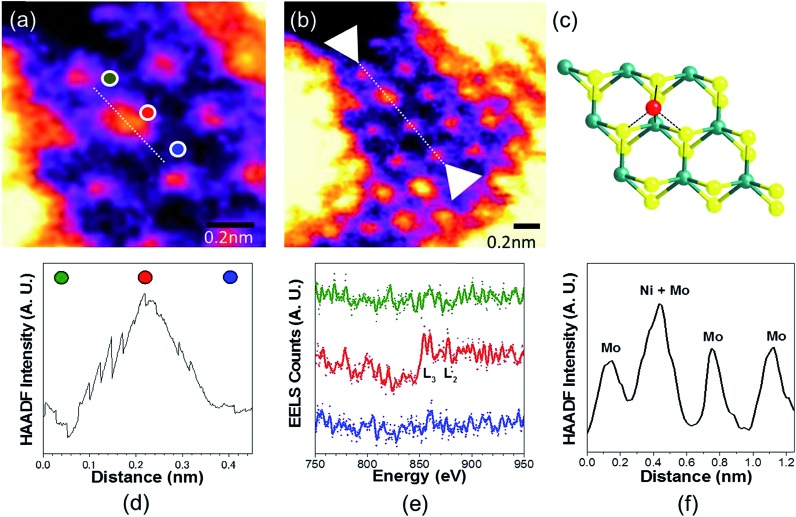
(a) and (b) HAADF-STEM images of Ni–^S^MoS_2_. (c) Ni at Mo-atop site model (d) ADF and (e) EELS acquired along the line in b. (f) ADF intensity line profile acquired along the line in b of the Mo sites in . (c) Ni at Mo-atop site model (d) ADF and (e) EELS acquired along the line in b. (f) ADF intensity line profile acquired along the line in b of the Mo sites in 〈100〉 direction.100. (c) Ni at Mo-atop site model (d) ADF and (e) EELS acquired along the line in b. (f) ADF intensity line profile acquired along the line in b of the Mo sites in 〈100〉 direction. direction.

To analyse the specific location of the two transition metal Co and Ni dopants, which show contrasting electrocatalytic properties on the basal plane of ^S^MoS_2_, High-Angle Annular Dark-Field (HAADF) imaging and Electron Energy Loss Spectroscopy (EELS) mapping of Co–^S^MoS_2_ (ref. 29) (Fig. 3) and Ni–^S^MoS_2_ (Fig. 4) were performed by High-Resolution Scanning Transmission Electron Microscopy (HR-STEM) and simulation (ESI, Fig. S4†). Both STEM analyses of the two samples show a characteristic and well-ordered hexagonal pattern of the honey-comb like structure which corresponds to monolayer ^S^MoS_2_. Due to the Z contrast nature of ADF imaging, Mo atoms (blue, Fig. 3a) would appear to be brighter than S atoms (yellow, Fig. 3a). In Fig. 3b, at least two major types of Co dwelling sites are clearly observed. First, a very bright image feature appears on top of the Mo position sandwiched by S atoms (white arrow). This is attributed to the single Co atom sitting on top of the trigonal prismatic Mo atom (Mo atop site).^29^ Similar features can also be observed at S positions (yellow arrow) (Fig. 3c and d), which can be attributed to the incorporation of a Co atom at the S vacant position (Co substituted in the S vacant site). For both samples, intensity profiles of the ADF image were taken across the doped sites in the ), which can be attributed to the incorporation of a Co atom at the S vacant position (Co substituted in the S vacant site). For both samples, intensity profiles of the ADF image were taken across the doped sites in the 〈110〉 direction and the corresponding atomic composition of the peaks were assigned (110), which can be attributed to the incorporation of a Co atom at the S vacant position (Co substituted in the S vacant site). For both samples, intensity profiles of the ADF image were taken across the doped sites in the 〈110〉 direction and the corresponding atomic composition of the peaks were assigned ( direction and the corresponding atomic composition of the peaks were assigned (Fig. 3e and f). ADF-EELS simultaneous acquisition line-scans were taken across a line containing either the single Co or Ni metal dopants (Co: Fig. 3e and f and Ni: Fig. 4d and e). For Co–^S^MoS_2_, the EELS extracted from before (green), on (dark blue), and after (khaki) the Co atom was doped on the Mo top site are shown in Fig. 3f. The characteristic L_3,2_ edges occur at the expected energy in the EELS extracted from Co on top of the Mo site, and are absent in the two other EELS. Both atop (Fig. 4c, e and f) and substituted S vacant sites can also be observed for Ni–^S^MoS_2_ (ESI, Fig. S5a and b†). The appearance of corresponding characteristic L_3,2_ edges at the original Mo position (red circle), compared to before (green) and after (blue), confirms the presence of the Ni atom (Fig. 4e and f). This is further supported by the ADF analysis along the Mo sites in the ). This is further supported by the ADF analysis along the Mo sites in the 〈100〉 direction, where the larger peak (red) is contributed by Ni doping on top of the Mo atom (Mo atop site). Although we occasionally detected the presence of the two metals by EELS on the edges of layers (see ESI, Fig. S6a, b and S7a, b100). This is further supported by the ADF analysis along the Mo sites in the 〈100〉 direction, where the larger peak (red) is contributed by Ni doping on top of the Mo atom (Mo atop site). Although we occasionally detected the presence of the two metals by EELS on the edges of layers (see ESI, Fig. S6a, b and S7a, b direction, where the larger peak (red) is contributed by Ni doping on top of the Mo atom (Mo atop site). Although we occasionally detected the presence of the two metals by EELS on the edges of layers (see ESI, Fig. S6a, b and S7a, b†), the large quantity and increasing number of basal sites with a significant increase in surface area compared to the peripheral edge sites of the reduced MoS_2_ slab size due to exfoliation make the metal doped basal sites a more dominant feature. Thus, the imaging results provide direct experimental evidence of the successful doping of a Co atom and a Ni atom onto the basal planes of our exfoliated samples of ^S^MoS_2_. Particularly, the chemisorption of the Co or Ni precursor directly on the abundant atop sites of the intact basal plane of ^S^MoS_2_ is envisaged to offer the accommodation of these transition metal atoms/ions in a significant quantity. It is also logical to assume that the Co or Ni precursor can occupy some surface S vacancies of ^S^MoS_2_ generated *via* the *n*BuLi exfoliation to establish Co–Mo or Ni–Mo interactions with the exposed Mo sites on the ruptured tri-atomic MoS_2_ layer. However, our STEM-EELS characterization so far was not able to offer any quantitative assessment of the main rooting sites for the two transition metals.

**Fig. 5:**
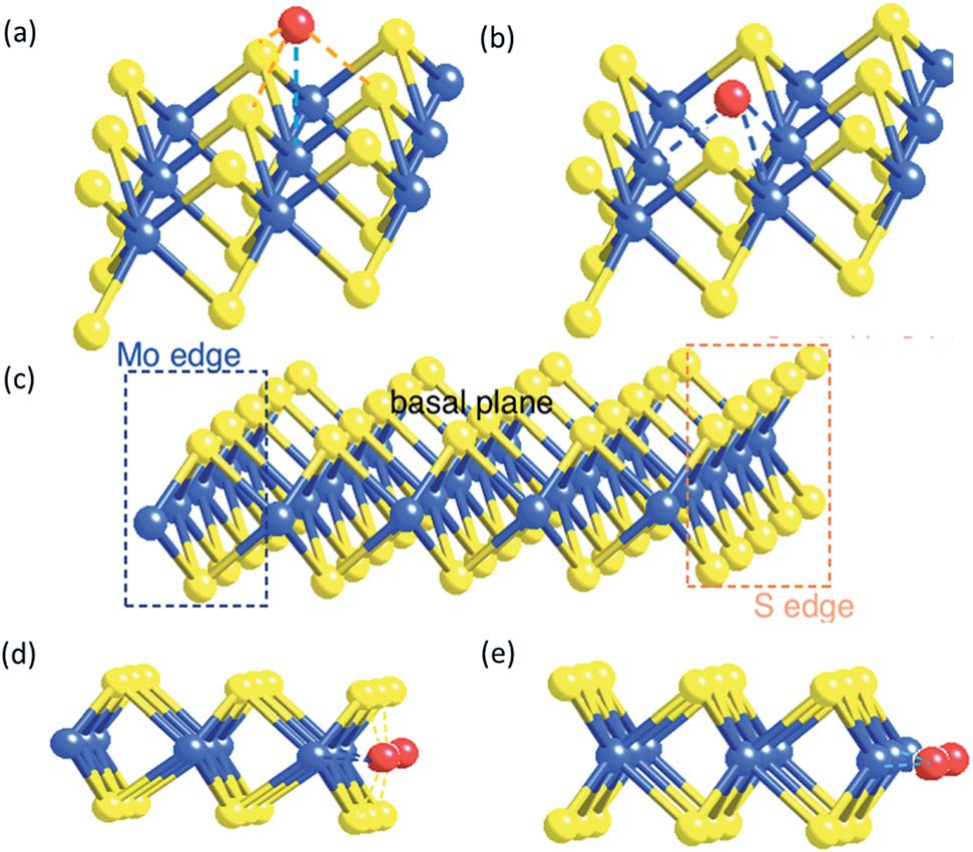
(a) A metal atom on the Mo top site of ^M^MoS_2_ (b) a metal atom on the S vacancy site of ^M^MoS_2_ (c) the monolayer of perfect triatomic MoS_2_ (d) metal doped S edge of ^M^MoS_2_ (e) metal-doped Mo edge of ^M^MoS_2_.

According to our molecular model of the single tri-atomic MoS_2_ layer, metal M on the Mo atop site of the basal plane of ^S^MoS_2_ (Fig. 5a) exerts somewhat similar geometric characteristics to form M–S interactions to that of the M doped S edge (Fig. 5d), as described in the S edge model (Fig. 5c). Similarly, when M substitutes at the S vacant site of the partially damaged basal plane of ^S^MoS_2_ (Fig. 5b), M–Mo interactions can be formed. This is akin to that of the M-doped Mo edge structure (Fig. 5e). Thus, the S vacancies on the monolayer MoS_2_ structure allow free access to the interior Mo sites modified by the chemisorbed M sulphide complex (akin to the Mo edge sites), which functions very similarly to the edge structure modification.

**Fig. 6:**
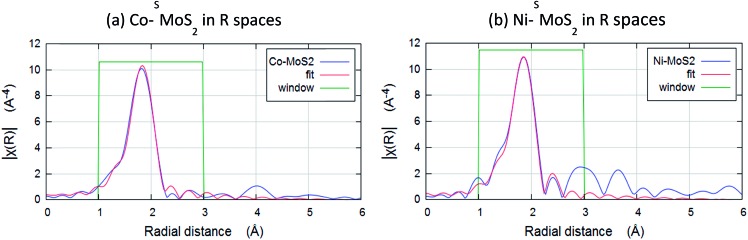
Fourier transforms of the k3-weighted Co and Ni K-edge of extended X-ray absorption fine structure spectroscopy (EXAFS) spectra of (a) Co–^S^MoS_2_ and (b) Ni–^S^MoS_2_.

The chemical environments of Co and Ni were then examined by Extended X-ray Absorption Fine Structure Spectroscopy (EXAFS). Fig. 6a and b show the result of Fourier transforms of the Co and Ni K-edge of Co–^S^MoS_2_ and Ni–^S^MoS_2_. All the parameters are fitted with acceptable Debye–Waller factors as listed in Table 1a and b. Both EXAFS results show there is no first-shell Co–Co or Ni–Ni contribution, suggesting that the major species of Co and Ni on the basal plane of the nanosheets are indeed not in the form of metal clusters nor nanoparticles but are single metal atoms/ions as suggested from the HADDF-STEM images (see Fig. 3 and 4). It is interesting to note that there is only a first shell Co–S contribution with a coordination number of about 4 (tetrahedron) at 2.27 ± 0.01 Å for Co–^S^MoS_2_ and a first shell Ni–S contribution with a coordination number of about 6 (octahedron) at 2.29 ± 0.01 Å for Ni–^S^MoS_2._ (Table 1). There is no first shell Co–Mo or Ni–Mo contribution, suggesting the majority of the Co and Ni species are indeed mainly on the atop sites (chemisorbed sites) of ^S^MoS_2_, since doping of M (Co or Ni) on the S vacancies or Mo edge sites to a significant extent would generate the corresponding first shell M–Mo interaction. Part of the first shell sulphide ligands of these two immobilized metal sulphides must be derived from the surface sulphide groups, presumably from the higher degree of exposure of the basal MoS_2_ plane during the synthesis. The higher affinity for octahedral coordinated 3d^8^ Ni^2+^ over tetrahedral coordinated 3d^7^ Co^2+^ with sulphide ligands due to the greater ligand field stabilization energy accounts for the fundamental difference in coordination environment.^38^ However, it is very interesting to find that the best fit data for Ni–^S^MoS_2_ depicts the presence of an Ni–Mo interaction at the second shell of 2.56 ± 0.04 Å with CN of 1.2 ± 0.3, whereas absolutely no Co–Mo interaction is detected in Co–^S^MoS_2_. Considering the larger size of Co (152 pm) and its tetrahedral coordination, the ‘Co–Mo’ distance between the shared triangular face with prismatic Mo (octahedron) is shorter than a typical Co–Mo bond from a simple geometric model. It therefore destabilizes the close face sharing for Co–Mo formation. On the other hand, the smaller size of Ni (149 pm) and its octahedral coordination can accommodate well for the stereospecific Ni–Mo interaction with the surface Mo site. The rigid close packed face-sharing model of chemisorbed transition metal sulphides can account for the absence of the Co–Mo interaction but facilities the Ni–Mo interaction for the same atop Mo site.

**Table 1:** EXAFS scattering path analysis of Co–^S^MoS_2_ and Ni–^S^MoS_2_

Scattering path	Bond length (Å)	Coordination number	*σ* ^2^
***R* = 0.9%, *K***_**wt**_ **= 1, 2, 3; *k* range 3–12; *R* range 1–3; *E***_**not**_ **0.23** ^*a*^			
Co–S	2.27 ± 0.01	3.9 ± 0.3	0.007

***R* = 1.8% *K***_**wt**_ **= 1, 2, 3; *k* range 3–12; *R* range 1–3; *E***_**not**_ **0.1** ^*a*^			
Ni–S	2.29 ± 0.01	6.5 ± 0.3	0.008
Ni–Mo	2.56 ± 0.04	1.2 ± 0.3	0.011

^*a*^ *E*_not_ is the difference in absorption energy between the experimental value and the calculated value.

### Density functional theory calculations

The favourability of incorporating a single metal atom M onto ^S^MoS_2_ is believed to cause the distinctive HER activity for electrochemical hydrogen production.

There have also been previous attempts to model the activity by doping single transition metal atoms onto the edge sites.^39^–^41^ The Gibbs free energy of hydrogen adsorption (Δ*G*_H_) is generally used as a descriptor for HER activity. It is believed that Δ*G*_H_ plays an important role in the electrochemical production of hydrogen gas: too weak H adsorption on the surface will not favour the electrochemical reaction and too strong H adsorption would also not facilitate H_2_ recombination and desorption. Thus, this theoretical value should be close to 0 to maximise the overall thermodynamic enthalpy value for the H surface adsorption, surface recombination and desorption processes in HER as previously discussed.^41^

**Fig. 7:**
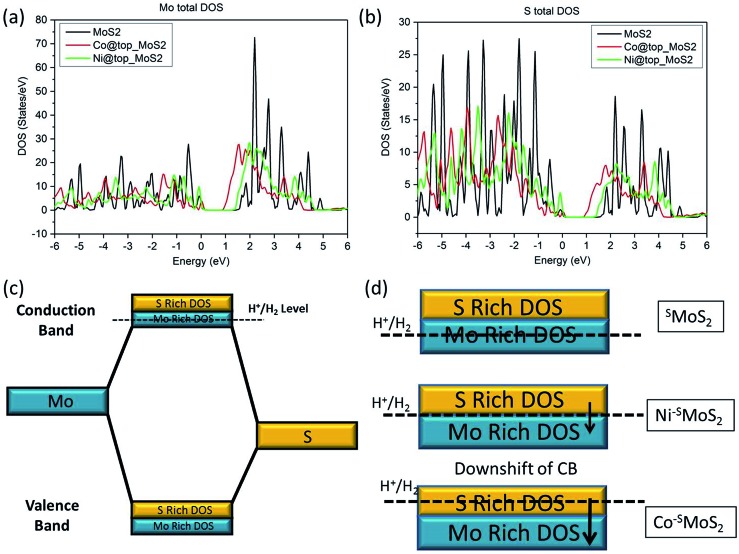
Calculated Density of States (DoS) of (a) Mo and (b) S in ^S^MoS_2_, Co–^S^MoS_2_ and Ni–^S^MoS_2_ (c) simplified molecular orbital diagram of MoS_2_ (d) downshift of the conduction band (CB) upon addition of a single Co and Ni metal atom at the Mo atop site, respectively.

The relationship between the rate of HER and the doping effect of the metal atoms at the Mo atop site on the basal plane can be rationalised with the help of the calculated density of states (DoS) spectra of the three MoS_2_ samples (^S^MoS_2_, Co–^S^MoS_2_, Ni–^S^MoS_2_) analysis (the Fermi energy level is arbitrarily set as 0, see ESI S1†). In general, the filled valance bands (VB) and the empty conduction bands (CB) in the DoS of MoS_2_ are composed of both S-3p and Mo-4d states (Fig. 7a and b). From Fig. 2b, we understand that hydrogen generation for all three MoS_2_ samples will progress through the Volmer–Heyrovsky mechanism. H^+^ reduction, as the rate determining step, takes place by promoting electrons from the VB to the empty CB by electrical means. For unmodified MoS_2_, the H^+^ reduction energy level (H^+^/H_2_) is close to the lower part of the CB, which is majorly occupied by the empty Mo-4d_*x*^2^–*y*^2^_ and Mo-4dz^2^ bands (ESI Fig. S8a to f†). Therefore, hydrogen will be mainly generated from the Mo sites with a small contribution from the S sites in pristine MoS_2_, as also described by the edge model.^39^ The value of the Gibbs free energy of hydrogen adsorption by the Mo component (Δ*G*_H–Mo_) in this case will be small and close to zero (Fig. 7d). It is expected that the addition of a 3d transition metal atom (Co and Ni) onto the Mo atop site will both cause a downshift on both the empty S-3p and Mo-4d CB bands (Fig. 7c). From our calculated DoS spectra, Co doping will induce a more remarkable downshift of the empty CB than that of Ni doping (Fig. 7a and b). Due to the absence of the Co–Mo interaction shown in our EXAFS results, it can be predicted that the H^+^ reduction energy level in Co–^S^MoS_2_ will now be very close to the upper level of the empty CB occupied by S-3p, thus the value of the Gibbs free energy of hydrogen adsorption by the S component (Δ*G*_H–S_) is close to zero (Fig. 7d). H^+^ reduction therefore mainly takes place over the S sites. For Ni–^S^MoS_2_, due to the smaller downshift of the CB as seen from our calculated bands (Fig. 7a and b), the H^+^ reduction level this time should be near the centre of the CB. While the contribution by both Mo and S to the overall H_2_ production are similar but not close to their individual S or Mo component centres, they give a smaller hydrogen contribution from both sites (Fig. 7d). This above model is consistent with the computational analysis of the change in Δ*G*_H_ due to edge site modification by Wang *et al.*^41^ Overall, due to the different degree of downshifting on both the S-3p and Mo-4d components in the CB as compared to ^S^MoS_2_, the Co atom selectively sitting at the Mo atop sites will enhance the HER activity while the Ni atom located on the same site with the stereospecifically allowed Ni–Mo interaction will show a much lower HER performance compared to undoped MoS_2_.

## Conclusions

In conclusion, we have provided direct experimental evidence that transition metal atoms can be chemically attached to multiple surface sites of basal plane of exfoliated ^S^MoS_2_. From the HADDF-STEM image and the EXAFS analysis the data suggests that the majority of the metal dopants are attached to the Mo atop sites on the basal ^S^MoS_2_ plane as chemisorbed sulphide complexes. For structural influence, it is difficult to use XRD to assess whether there is any change in the monolayer MoS_2_ structure due to the doped atoms since our samples did not give good quality peaks for the structural analysis. Instead, our STEM images, EXAFS analysis and Raman spectra (given in the revised ESI†) suggested that there was no significant change in the crystallographic parameters of the underneath monolayer of MoS_2_ (*i.e.* the Mo–S, Mo–Mo, and S–S distances). However, the anchored tetrahedral coordinated Co can affect the surface neighbour S sites through a Co–S interaction on the basal plane in attenuating Δ*G*_H_ towards a value of zero at high hydrogen coverage hence enhancing the HER activity. On the other hand, the geometric constraints of the large sized Co atom in the tetrahedral arrangement deny the direct influence of the Mo site on ^S^MoS_2_ with no Co–Mo interaction. In contrast, the chemisorbed octahedral Ni sulphide on the same atop site allows the direct electronic modification of the Mo site by establishing a Ni–Mo interaction, which causes the deviation of Δ*G*_H_ from zero at the surface exposed Mo sites at high hydrogen coverage hence greatly attenuating the HER activity. As seen from the DFT calculations, we have also attributed their difference in HER activity to the electronic modifications of the MoS_2_ structure by the doped atoms/ions. Thus, both the structural and electronic factors are expected to influence their resulting activity over the single atom doped monolayer MoS_2_ samples. It is believed that both the geometric and electronic factors exerted by the transition metal dopants are therefore important parameters in further tuning the 2D MoS_2_ structure for the rational design of composite materials for more efficient electrochemical hydrogen production from water.

## Contributions

THML, XL and JK prepared the samples and carried out testing; SW, TW and YS worked on the XAS (EXAFS); RK and KS did the STEM and EELS; LL carried out the DFT calculations; JSF advised on the electrochemical measurements; THML and SCET wrote the paper, SCET planned and supervised this project. All contributed toward the preparation of the manuscript.

## Conflicts of interest

There is no conflict of interest to declare.

## Supplementary Material

Supplementary information
