# Supplementary material for: Transition metal atom doping of the basal plane of MoS2 monolayer nanosheets for electrochemical hydrogen evolution
Source: Chem Sci. 2018 Apr 30;9(21):4769–76. doi: 10.1039/c8sc01114a (PMC5975547; doi:10.1039/c8sc01114a)
Supplement: Supplementary file 1 [file SC-009-C8SC01114A-s001.pdf]

Electronic Supplementary Material (ESI) for Chemical Science.
This journal is © The Royal Society of Chemistry 2018

Transition Metal Atoms Doping on Basal Planes of MoS_2 Monolayer Nanosheets for Electrochemical Hydrogen Evolution

Thomas H.M Lau, Xiao Wei Lu, Jiří Kulhavý, Simson Wu, Lilin Lu, Tai-Sing Wu, Ryuichi Kato, John S. Foord, Yun-Liang Soo, Kazu Suenaga and Shik Chi Edman Tsang

Supplementary Information

S1 Synthesis of Few-Layered MoS_2 ($^{F}MoS_2$) and Single-Layered MoS_2 ($^{S}MoS_2$)

Few-Layered MoS_2. 6g of bulk MoS_2 powder was dispersed in 400mL of Water/Isopropanol (1:3, v/v). 4 mL of hydrazine monohydrate was then added. The solution mixture was placed into the sonication bath for 12 hours for exfoliation, followed by centrifugation at 2000 rpm for 60 minutes. The supernatant collected was filtered using vacuum filtration, followed by washing with water. The exfoliated product was dried under vacuum for 24 hours.

Single-Layered MoS_2. 0.5g of bulk MoS_2 powder was soaked in 4mL of 1.6M n-butyllithium/hexane under nitrogen atmosphere for 48 hours. Solid Li_xMoS_2 was then isolated by vacuum filtration, followed by washing with hexane to remove excess n-butyllithium. It was then dried under vacuum for 24 hours. The dried product was then immersed into 250mL of water. The solution was placed into the sonication bath for 12 hours and then centrifuged at 5000 rpm for 15 minutes. The supernatant collected was filtered using vacuum filtration, followed by washing with water. The exfoliated product was dried under vacuum for 24 hours.

Synthesis of Single Transition-Metal Atom Doped $^{F}MoS_2$/$^{S}MoS_2$

5 different transition-metals (Ag, Co, Ni, Pd, Ru) were used for doping in this paper. Each precursor solution was prepared by dissolving 0.2mM Metal ions into 1mL of 0.5mM thiourea solution and left for overnight to form a metal complex. The metal complex solution was mixed with 30mL of colloid solution, which is made by dispersing 30mg of $^{S}MoS_2$ (or $^{F}MoS_2$) in 30mL of Water/Isopropanol (1:3, v/v) and 15 μL of 5 wt% Nafion®117 solution (Stabiliser). The solution mixture was then transferred to an autoclave and then placed into the oven at 160°C for 24 hours. Afterwards, the precipitate was washed with deionized water and dried under vacuum for 12 hours to obtain the solid product.

S2. Characterization

Scanning transition electron microscopy-electron energy loss spectroscopy (STEM-EELS). STEM-EELS was performed using a JEOL 2100F TEM with a DELTA aberration corrector operated at 60 kV and a GIF Quantum for spectroscopy. The specimen was heated to 300°C using an in-situ high temperature sample holder to help prevent surface contamination, and was left in the microscope column overnight before data acquisition so as to stabilise. The ADF images were acquired by using an inner acquisition semi-angle of 79 mrad and a convergence semi-angle of 35 mrad. ADF-STEM images were subjected to a 4 Gaussian smoothing filter (2px), and EELS profiles were smoothed with a Savitsky-Golay algorithm. ADF image simulations were performed using microscope parameters corresponding to the experimental conditions (4 nm defocus, Cs 2 μm, 37 nm three-fold astigmatism, 30 nm coma, and 35 mrad half convergence angle). Low-voltage, low-current and short exposure duration were used to reduce any beam damage to the material3. The high-angle scattered electrons that are captured to form the ADF image are strongly sensitive to atomic weight, allowing for Z-contrast imaging.

Electrochemical Measurement. A three-electrode system controlled by μ-AUTOLAB III potentiostat (Eco-Chemie, Netherlands) was applied to carry out all electrochemical measurements. A platinum coil counter electrode and an Ag|AgCl ($1 molL^{-1} KCl$) reference electrode were used in this study. To prepare the working electrode, 4 mg of the electrocatalyst and 80μL of 5 wt% Nafion®117 solution will be dispersed in 1 ml of water/ethanol (4:1 v/v) and then followed by 30 min sonication to create a homogeneous ink. The ink was then drop-coated onto the surface of a glassy carbon electrode with a surface diameter of 3mm. All measurements were calibrated with respect to the reversible hydrogen electrode (RHE). Several precautions for minimising the IR drop effect were taken. The electrolyte (0.5M H_2SO_4) was used as it provided sufficient ions to keep the solution resistance low and the glassy carbon working microelectrode was fairly conductive and small in surface area. While it was difficult to obtain perfect cell geometry, a standard electrochemical cell was used to keep the distance between three electrodes fixed. The maximum current measured was in a magnitude of 10^{-3} A, which was quite low when comparing to that of a real cell setting. The IR compensation circuit fitted with a digital-to-

analog converter was connected to the potentiostat, which helped the partially compensation for the IR drop during the electrochemical scans.

S3. Others

Oxygen impurities

Xie *et al.* have verified that oxygen can be incorporated into MoS_2 ultrathin nanosheets when the temperature of the hydrothermal reaction is low[1]. As a result of insufficient energy, some of the Mo-O bonds from $(NH_4)_6Mo_7O_{24}\cdot 4H_2O$ precursors remained in the final product as impurities. In our experiment, we adopted the top-down exfoliation method from bulk crystalline MoS_2 rather than the bottom-up hydrothermal synthesis. No other oxygen sources were added throughout the experiment. While it was still possible to create oxygen impurities during the hydrothermal doping process due to the presence of atmospheric oxygen, we did not detect any oxygen signal from EDX and EELS, indicating that the amount of oxygen impurities must be comparatively small. In addition, the working electrode had been cycled for multiple times to remove any possible oxygen impurities on the catalyst surface before conducting the linear sweep voltammetry for HER reaction.

Previous Works on Co-$^{S}MoS_2$

The previous work from our research group[2] studied particularly on Co-$^{S}MoS_2$ for hydrodeoxygenation reaction. The synthesis of Co-MoS_2 for thermocatalytic hydrodeoxygenation reaction in our previous work required extra annealing step at 300°C with H_2 for 1 h after the exfoliation process to generate additional vacancies for this higher temperature reaction. Co on Mo atop sites, Co in S substitution sites and Co in hollow sites were all seen in this case due to the higher activation energy used and the additional H_2 removal of some S sites, etc. In addition, we can see the direct Co-Mo interaction in such case[2]. However, in this manuscript, no annealing step in H_2 at high temperature was used for this HER activity study of the Co-MoS_2 and only two sites, namely, only Co on Mo atop sites and Co in S substitution sites were mainly observed. For the samples, after the HER reaction we did not see any new configuration occurred. In addition, from our experimental results, the Co-MoS_2 gave high stability despite several hundreds of CV cycles as compared to its initial cycle where EXAFS showed the principal Co dwell site is in Mo atop position. If the Co atoms moved into different sites during the HER reactions, we would expect that the HER activities should have given a noticeable change.

Turnover frequencies

The expression of turnover frequencies of Co-MoS_2 or Ni-MoS_2 for their dwelling sites would be extremely useful for researchers to compare with the other most-of-the-art single atom catalyst for HER. Although the overall Co concentration (3 wt%) on the monolayer MoS_2 structure is known, it is however, challenging to achieve the expression at present due to inability to assess quantitatively the number of Co atoms on the atop and S substituted sites versus some edge sites by the STEM. The average density may be estimated but is still very challenging to do the STEM in statically reliable manner. Besides, insufficient literature data is available for a specific TOF for this HER reaction can be compared so far.

S4 Additional Figures

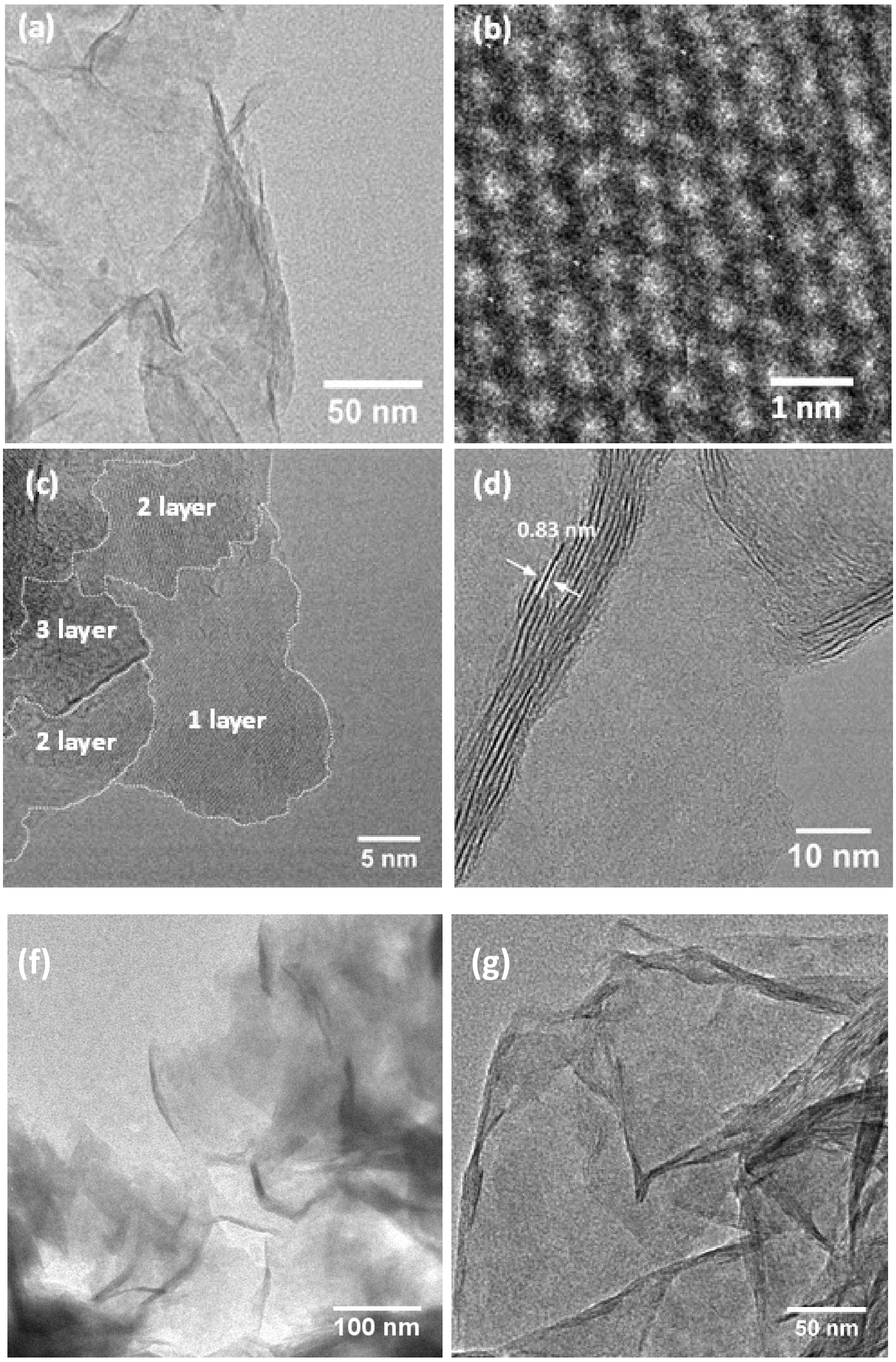

Figure S1 **a,** Sheet- like morphology exfoliated $^{S}MoS_2$. HRTEM images of **b,** typical structure of the MoS_2. **c,** Top view and **d,** side view of restacked $^{S}MoS_2$. TEM images of **f**, Co-$^{s}MoS_2$ and **g**, Ni-$^{s}MoS_2$. While minor agglomeration and restacking may have occurred after the hydrothermal reaction of doping of transition metal on MoS_2, most areas of $^{S}MoS_2$ with the metal doping as seen from images **f** and **g** still show a sheet-like morphology.

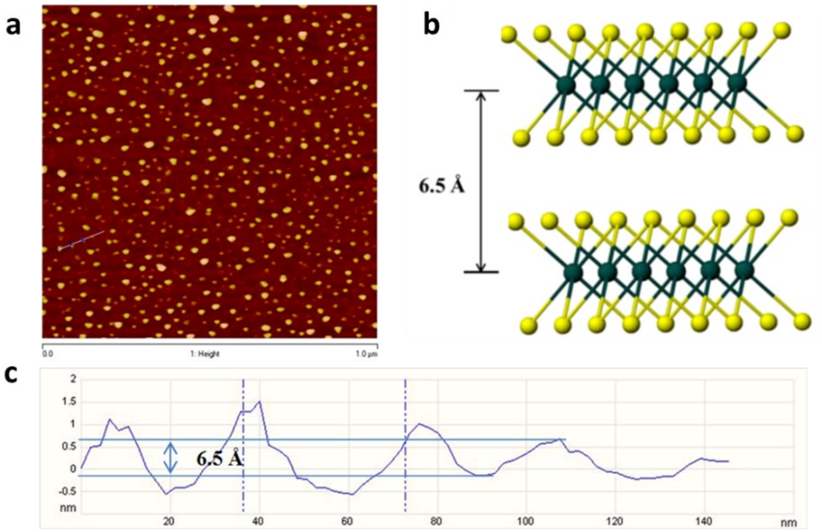

Figure S2. Atomic force microscopy (AFM) image analyses for the fresh chemically exfoliated $^{S}MoS_2$. **a**, AFM image of spin-coated $^{S}MoS_2$ and **b**, a model of 2-H MoS_2 structure perpendicular to c axis, 100 flakes is scanned with majority of heights between 0.6-0.7nm. The sample was prepared by spin-coating $^{S}MoS_2$ onto a surface of Si/SiO_2 substrate. The lateral dimension of this $^{S}MoS_2$ nanosheet is approximately 20-40 nm. **c**, It can be seen that the step heights of individual layers of 0.6-0.7 nm. This value is comparable to ca. 0.65 nm of a single layer of the S-Mo-S building block. Statistical analysis of 100 flakes produced by the lithium exfoliation method revealed that 56% of the flakes to be monolayer, 28% of two layers and 13% of three layers and so on. The average topographic height is around 1.04 nm, which agrees with typical height of a $^{S}MoS_2$ with the presence of water molecules (between 0.6 and 1.0 nm).

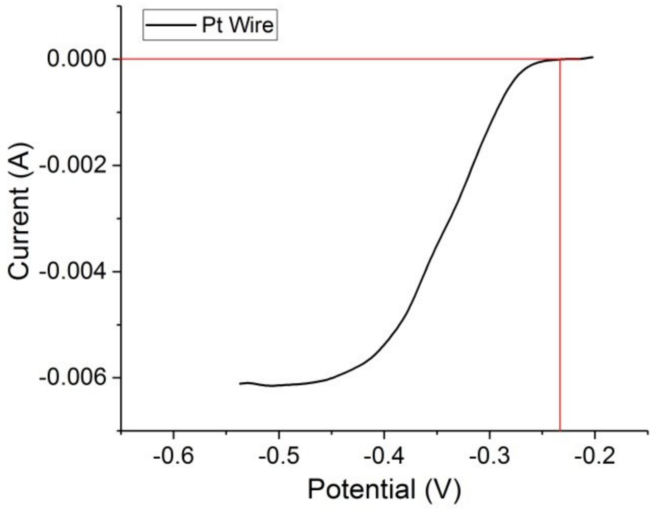

Figure S3a. Calibration plot of reference electrode Ag|AgCl with respect to RHE in H_2-saturated 0.5 M H_2SO_4. All the potentials referenced to the reversible hydrogen electrode (RHE) have added a value of 0.235 V during all the analyses. ($E_{RHE} = E_{Ag|AgCl} + 0.235V$)

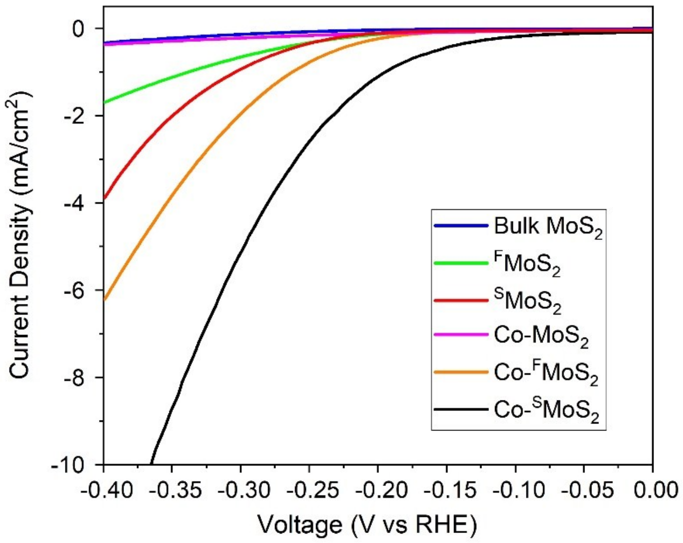

Figure S3b. LSV of MoS_2 nanosheets with different thickness in 0.5M H_2SO_4 at a scan rate of 2 mVs^{-1}.

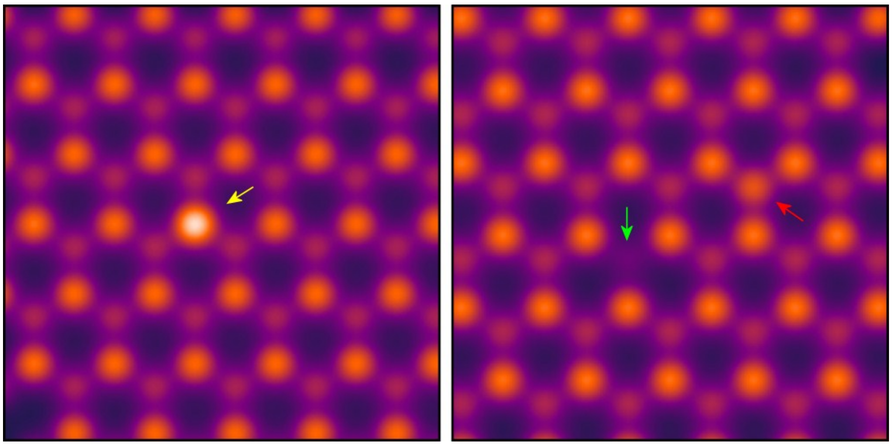

Figure S4 STEM image simulations. Yellow arrow shows M on Mo, green a single S vacancy, and red, a M substituted for S.

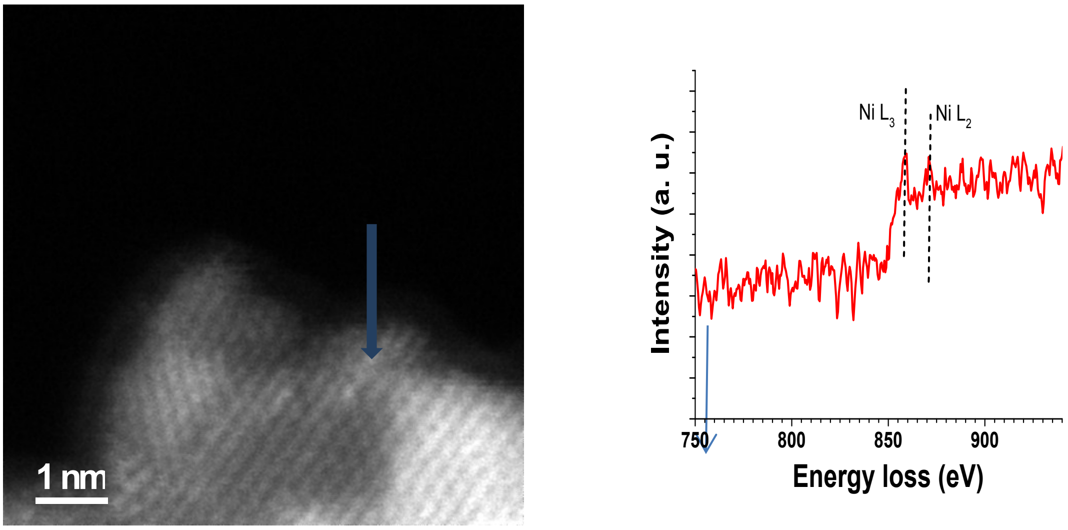

Figure S5 **a**, left: A Ni substituted S vacant site in exfoliated $^{S}MoS_2$. **b**, right: EELS acquired along the line in **a**.

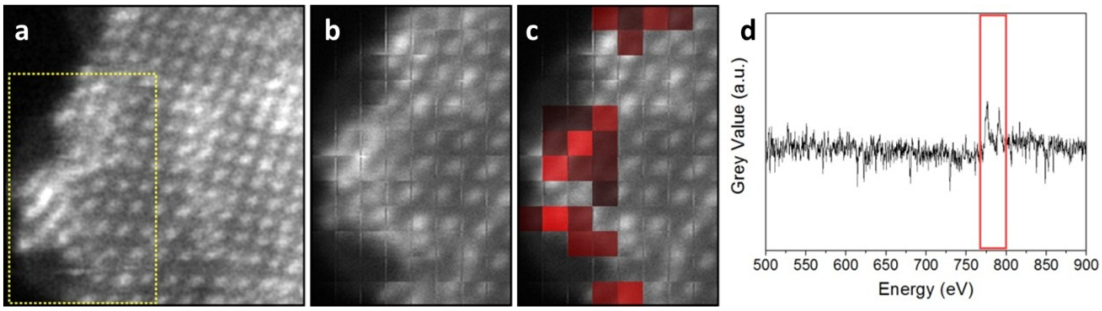

Figure S6 **a,** ADF image of the edge of Co-$^{S}MoS_2$ sample. **b** and **c,** ADF-STEM and EELS intensity mapping from the boxed region in (**a**). **d,** EELS extracted from a 3 × 3 bin from (**c**) showing the $L_{2,3}$ Co peaks. The red box indicates the energy range over which the intensity mapping in (**c**) is taken. The simultaneous acquisition ADF and EELS map from the edge sites of Co-$^{S}MoS_2$, with red strength confirming the presence of Co atoms from the EELS integrated intensity in the energy window 770–800 eV (Co $L_{3,2}$ edge).

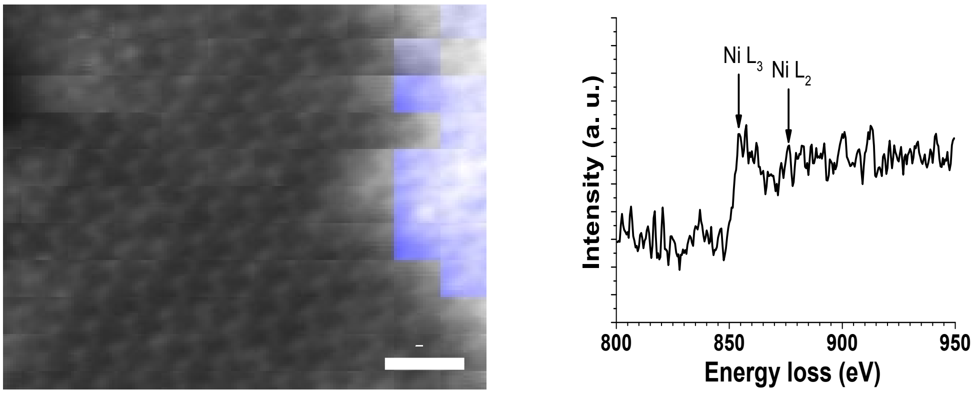

Figure S7 **a,** ADF image and EELS intensity mapping from the edge region. **b,** EELS extracted from a 3 × 3 bin from (**a**) showing the $L_{2,3}$ Ni peaks. The Ni is located at edge of multilayer MoS_2, easy to contamination of hydrocarbons in the equipment.

Table S1. A typical result of Inductive coupled plasma mass spectrometry (ICP-MS) of 5.043mg Co-MoS_2

Mo				Co			
Wavelength (nm)	Intensity	Concentration[b] （mg/L）	Content[c] (mg/mg)	Wavelength (nm)	Intensity	Concentration （mg/L）	Content (mg/mg)
202.03	1176703	51.75	0.256543724	228.615	466868	5.69	0.028207416
277.54	1884379	52.41	0.259815586	236.379	393049	5.89	0.02919889
202.03	1196678	52.63	0.260906207	228.615	473938	5.77	0.033408208
277.54	1921969	53.46	0.265020821	236.379	397359	5.95	0.029496332

DFT calculation details

Electronic structure calculations and geometrical optimizations were performed using density-functional theory as implemented in Vienna ab initio simulation packages (VASP) [G. Kresse, J. Hafner, Phys. Rev. B 1993, 47, 558-561. G. Kresse and J. Furthmuller, Phys. Rev. B: Condens. Matter Mater. Phys., 1996, 54, 11169-11186.], the exchange-correlation energy was calculated using Perdew-Burke-Ernzerhof (PBE) functional [J. P. Perdew, K. Burke and M. Ernzerhof, Phys. Rev. Lett., 1996, 77, 3865-3868.], and the ion-electron interaction was treated using the projector-augmented wave (PAW) method [P. E. Blochl, Phys. Rev. B: Condens. Matter Mater. Phys., 1994, 50, 17953-17979.], the cutoff of plane wave basis was set to 400 eV for solving the Kohn-Sham equations [W. Kohn, L. J. Sham, Phys. Rev., 1965, 140, A1133-A1138.]. A (3×3) supercell of 2H-MoS_2 was selected to simulate the monolayer molybdenum disulfide ($^{S}MoS_2$) basal plane, periodic boundary conditions were used and 15 Å of vacuum in the *z*-direction was used to separate neighboring MoS_2 basal planes. The Brillouin zone has been sampled using an 2×2×1 and a 8×8×4 Monkhorst-Pack [H. J. Monkhorst and J. D. Pack, Phys. Rev. B: Solid State, 1976, 13, 5188-5192.] grid of k-points for geometry optimizations and density of states (DOS) calculations, respectively. Both lattice constants and atomic positions were relaxed until the forces on atoms were less than 0.02 eV $Å^{-1}$ and the total energy change was less than $1.0×10^5$ eV.

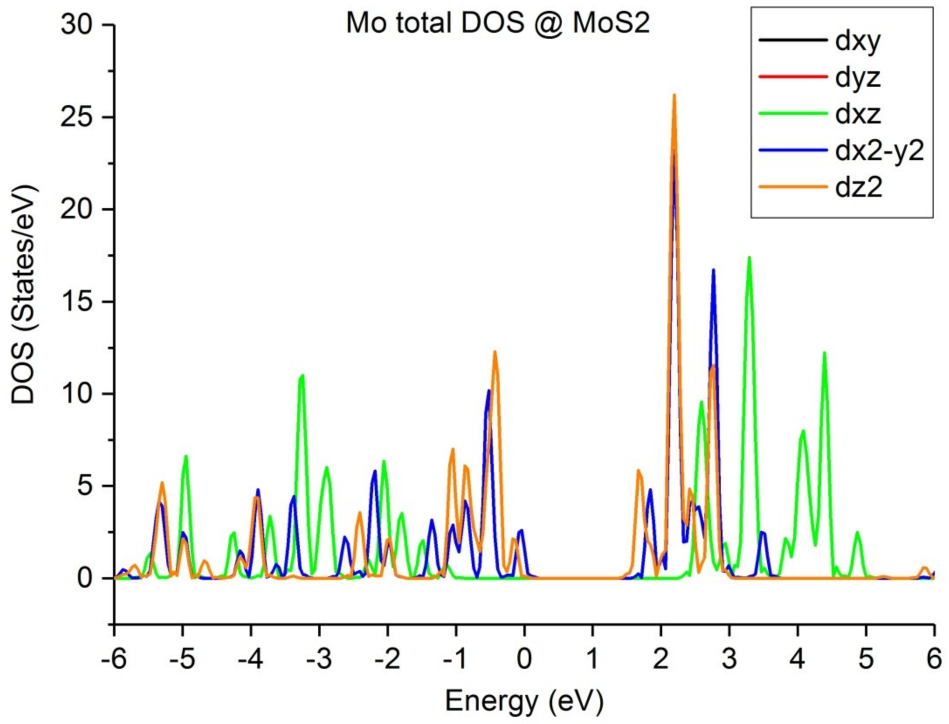

Figure S8a. DOS breakdown spectrum of Mo of MoS_2.

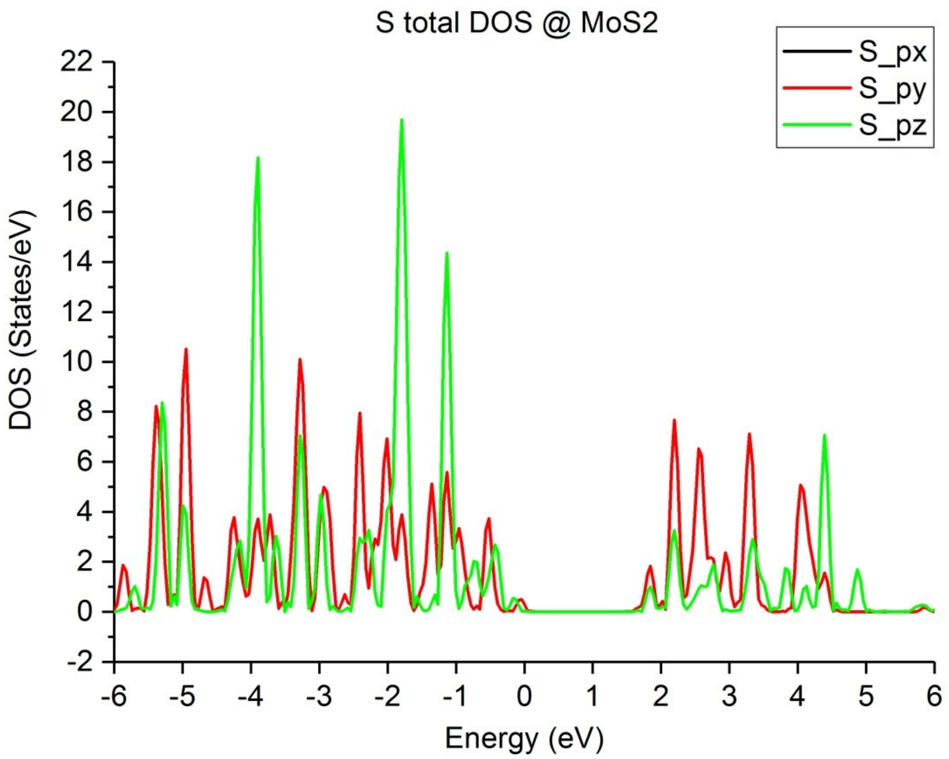

Figure S8b. DOS breakdown spectrum of S of MoS_2.

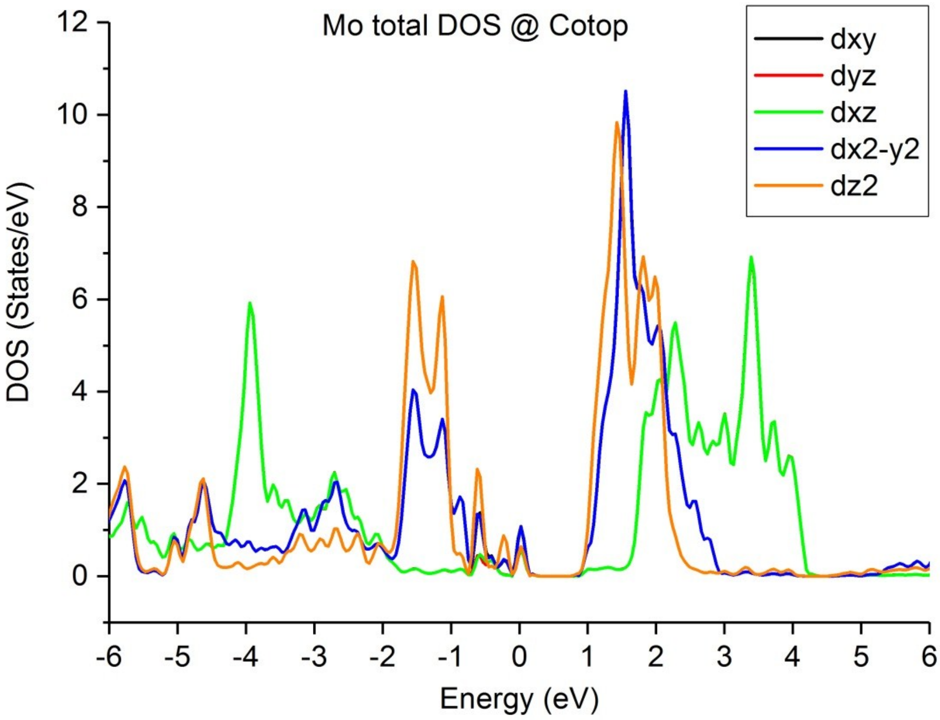

Figure S8c. DOS breakdown spectrum of Mo of Co-$^{S}MoS_2$ with Co doped at Mo atop sites.

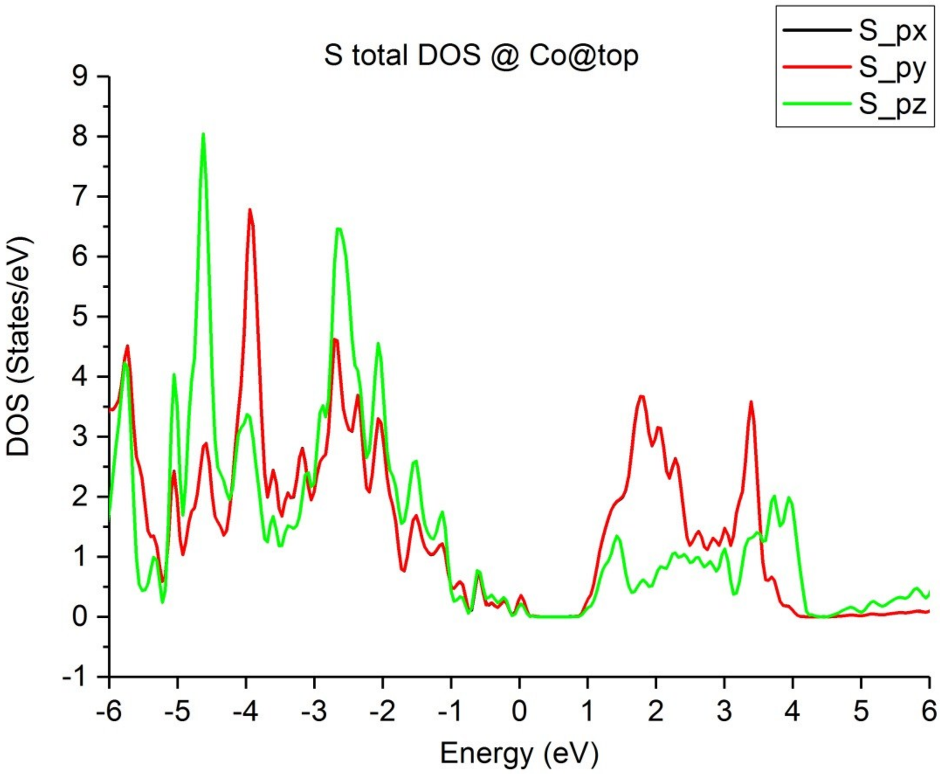

Figure S8d. DOS breakdown spectrum of Mo of Co-$^{S}MoS_2$ with Co doped at Mo atop sites.

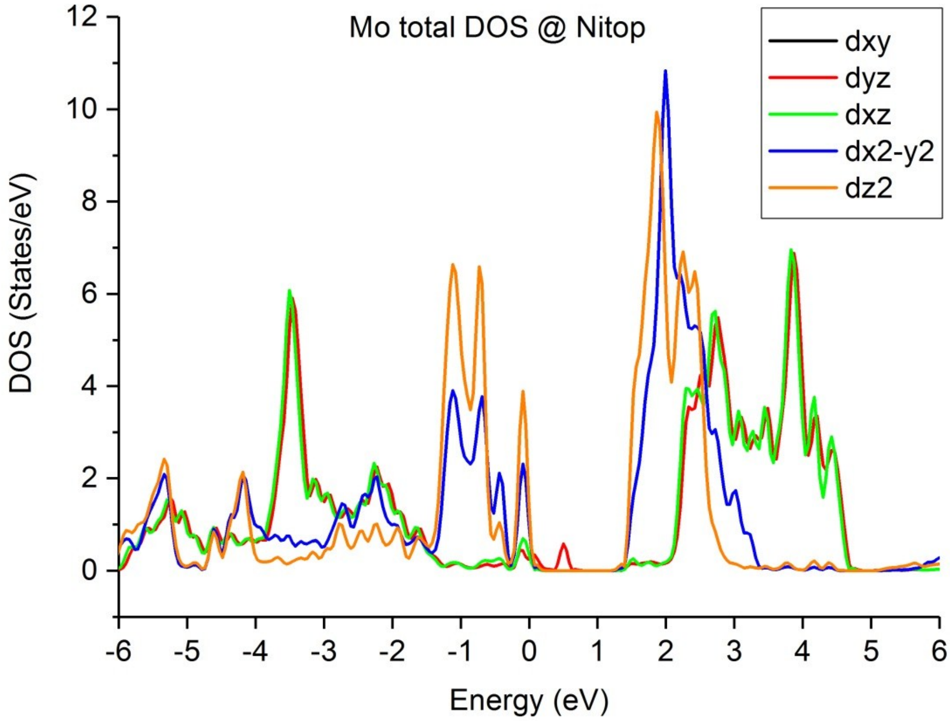

Figure S8e. DOS breakdown spectrum of Mo of Ni-$^{S}MoS_2$ with Ni doped at Mo atop sites.

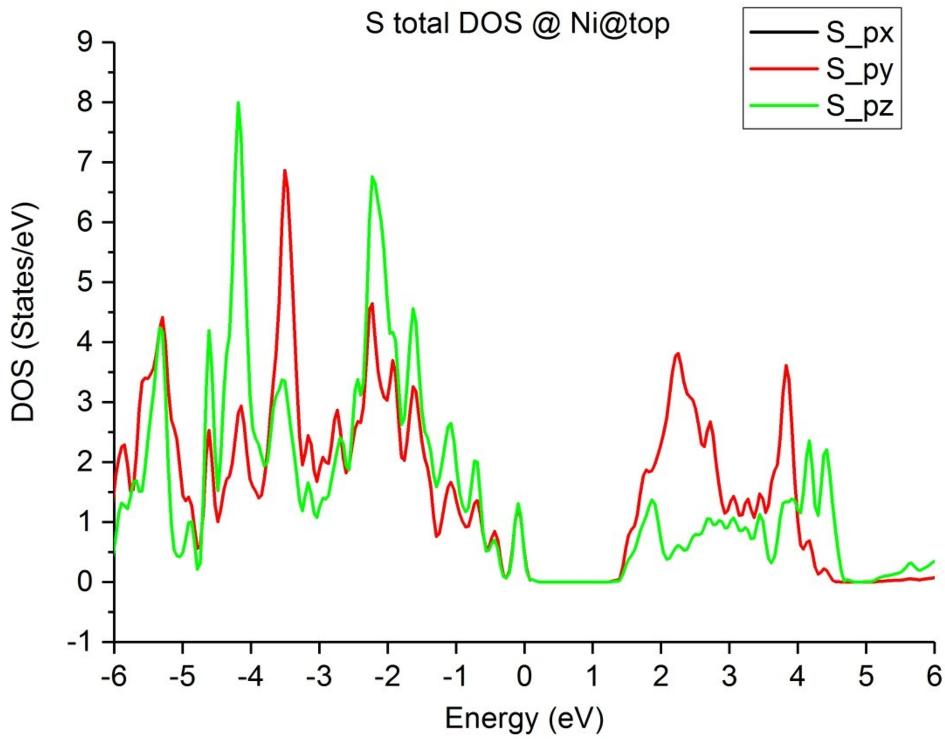

Figure S8f. DOS breakdown spectrum of S of Ni-$^{S}MoS_2$ with Ni doped at Mo atop sites.

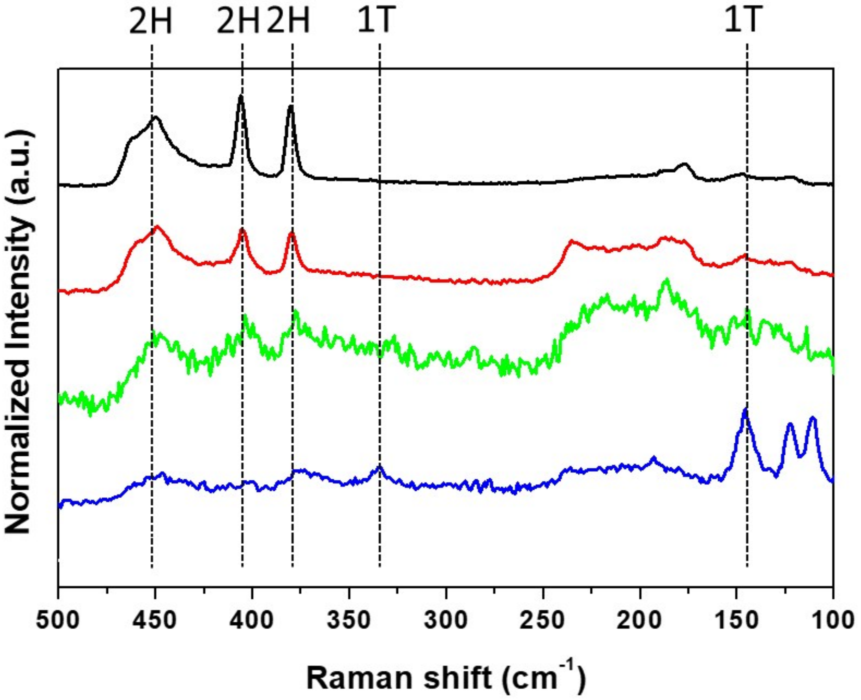

Figure S9. Raman spectrum of Bulk MoS_2 (black), $^{F}MoS_2$ (red), Co-$^{S}MoS_2$ after hydrothermal synthesis (Green), and fresh $^{S}MoS_2$ (blue), respectively. 1T phase of fresh $^{S}MoS_2$ is known to be metastable and can be rapidly reverted back to the stable 2H phase upon heating at around 100°C.[3,4] Since the hydrothermal reaction for Co doping on fresh $^{S}MoS_2$ (showing the 1T feature) has been taken place at 160°C for prolonged time, and thus the resulted Co-$^{S}MoS_2$ after the hydrothermal synthesis seems to have converted to the 2H phase as shown above.

References

1 J. Xie, J. Zhang, S. Li, F. Grote, X. Zhang, H. Zhang, R. Wang, Y. Lei, B. Pan and Y. Xie, *J. Am. Chem. Soc.*, 2013, **135**, 17881–17888.

2 G. Liu, A. W. Robertson, M. M.-J. Li, W. C. H. Kuo, M. T. Darby, M. H. Muhieddine, Y.-C. Lin, K. Suenaga, M. Stamatakis, J. H. Warner and S. C. E. Tsang, *Nat. Chem.*, 2017, **9**, 810–816.

3. P. Cheng, K. Sun , Y. H. Hu, RSC Adv., 2016,**6**, 65691-65697.

4. G. Eda, H. Yamaguchi, D. Voiry, T. Fujita, M. Chen, M. Chhowalla, *Nano Lett.*, 2011, **11** (12), 5111–5116.
